# Using School Staff Members to Implement a Childhood Obesity Prevention Intervention in Low-Income School Districts: the Massachusetts Childhood Obesity Research Demonstration (MA-CORD Project), 2012–2014

**DOI:** 10.5888/pcd14.160381

**Published:** 2017-01-12

**Authors:** Rachel E. Blaine, Rebecca L. Franckle, Claudia Ganter, Jennifer Falbe, Catherine Giles, Shaniece Criss, Jo-Ann Kwass, Thomas Land, Steven L. Gortmaker, Emmeline Chuang, Kirsten K. Davison, MA-CORD Project Group

**Affiliations:** 1 Department of Family and Consumer Sciences, California State University, Long Beach, California; 2 Department of Nutrition, Harvard T.H. Chan School of Public Health, Boston, Massachusetts; 3 Medical Faculty Mannheim, Heidelberg University, Mannheim Institute of Public Health, Social and Preventive Medicine, Mannheim, Germany; 4 University of California, Berkeley, Berkeley, California; 5 Department of Social and Behavioral Sciences, Harvard T.H. Chan School of Public Health, Boston, Massachusetts; 6 Health Sciences Department, Furman University, Greenville, South Carolina; 7 Bureau of Community Health and Prevention, Massachusetts Department of Public Health, Boston, Massachusetts; 8 Office of Data Management and Outcomes Assessment, Massachusetts Department of Public Health, Boston, Massachusetts; 9 University of California, Los Angeles, Fielding School of Public Health, Los Angeles, California.

## Abstract

**Introduction:**

Although evidence-based interventions to prevent childhood obesity in school settings exist, few studies have identified factors that enhance school districts’ capacity to undertake such efforts. We describe the implementation of a school-based intervention using classroom lessons based on existing “Eat Well and Keep Moving” and “Planet Health” behavior change interventions and schoolwide activities to target 5,144 children in 4th through 7th grade in 2 low-income school districts.

**Methods:**

The intervention was part of the Massachusetts Childhood Obesity Research Demonstration (MA-CORD) project, a multisector community-based intervention implemented from 2012 through 2014. Using mixed methods, we operationalized key implementation outcomes, including acceptability, adoption, appropriateness, feasibility, implementation fidelity, perceived implementation cost, reach, and sustainability.

**Results:**

MA-CORD was adopted in 2 school districts that were facing resource limitations and competing priorities. Although strong leadership support existed in both communities at baseline, one district’s staff reported less schoolwide readiness and commitment. Consequently, fewer teachers reported engaging in training, teaching lessons, or planning to sustain the lessons after MA-CORD. Interviews showed that principal and superintendent turnover, statewide testing, and teacher burnout limited implementation; passionate wellness champions in schools appeared to offset implementation barriers.

**Conclusion:**

Future interventions should assess adoption readiness at both leadership and staff levels, offer curriculum training sessions during school hours, use school nurses or health teachers as wellness champions to support teachers, and offer incentives such as staff stipends or play equipment to encourage school participation and sustained intervention activities.

## Introduction

Childhood obesity threatens the health of American children, especially those in low-income households (1,2). Although evidence supports the efficacy of school-based interventions in reducing obesogenic behaviors and body mass index (BMI) among children (3–6), limited data describe school districts’ capacity to undertake such interventions (7). In 2011, the Centers for Disease Control and Prevention funded 4 grantees to conduct a 4-year Childhood Obesity Research Demonstration (CORD) project aimed at improving low-income children’s nutrition and physical activity behaviors. This study describes the implementation of a school-based obesity prevention intervention within the Massachusetts CORD project (MA-CORD) in 2 low-income school districts (8). Using a mixed methods design, we assessed facilitators and barriers to achieving implementation outcomes adapted from the taxonomy of Proctor et al (9). We hypothesized that a classroom-based health behavior intervention for 4th through 7th grade students would be most effective when the school staff felt activities were appropriate, feasible, and supported by district administrators.

Examining implementation outcomes (eg, extent to which an intervention is adopted by teachers) provides context for intervention outcomes (eg, change in children’s BMI) and is needed to ensure that interventions are effectively adopted, translated, and sustained in community settings. Implementation outcomes can also serve as proximal indicators of intervention outcomes, which are described elsewhere (10). We provide an overview of MA-CORD adoption, implementation, and potential to be sustained, along with a summary of strategies for remediating implementation barriers.

## Methods

MA-CORD was a multilevel, multisector intervention to prevent or control obesity among children aged 2 to 12 years in 2 low-income communities (mean annual per capita income <$35,000) in Massachusetts with greater-than-average prevalence of childhood obesity (combined mean, 26%) relative to national estimates (17%) (10). Community 1’s population of approximately 40,000, and Community 2’s population of approximately 95,000 each has a single school district. MA-CORD was implemented from 2012 through 2014 across 6 sectors (health care; early childhood care and education; school; afterschool; Women, Infants, and Children [WIC]; and the broad community). MA-CORD targeted obesity-related behaviors: fruit and vegetable consumption, sugar-sweetened beverage consumption, physical inactivity, screen time, and insufficient sleep duration and quality. Detailed information on MA-CORD intervention components is published elsewhere (8,10).

The MA-CORD school intervention consisted of evidence-based components: teacher training, curriculum delivery, use of wellness champions (eg, school nurses, teachers), provision of physical activity supplies (eg, balls, jump ropes), and educational materials (eg, flyers, banners). Each district used one part-time, paid coordinator to oversee administration of MA-CORD. Wellness champions were identified at baseline in each school and compensated $1,000 per academic year to lead school-wide wellness activities (eg, improved policies, fun runs, student media competitions) that reinforced MA-CORD messages and classroom interventions. School nurses received $500 per academic year to support MA-CORD data collection and wellness activities.

We focused on the role of teachers in administering adapted versions of evidence-based interventions designed for students in 4th and 5th grade elementary school (*Eat Well and Keep Moving*) and 6th and 7th grade middle school (*Planet Health*) (3,4). In year 1, teachers received a 3-hour training that introduced curricula materials to be integrated across major subjects (ie, math, language arts, and social studies). In Community 1, teachers were trained during school hours, and MA-CORD funds supplied substitute teachers for the time. In Community 2, teachers were trained after school hours and compensated $100. Teachers were encouraged to incorporate at least 6 lesson plans aligned with MA-CORD behavioral targets per academic year. In lieu of training all classroom teachers, Community 1 administrators opted to train health education teachers exclusively to implement the lessons across grades 4 through 7. Because each health teacher taught multiple classes across grades, this meant fewer teachers required training. In Community 2, both classroom teachers (grades 4 and 5) and health teachers (grades 6 and 7) received training.

**Figure 1:**
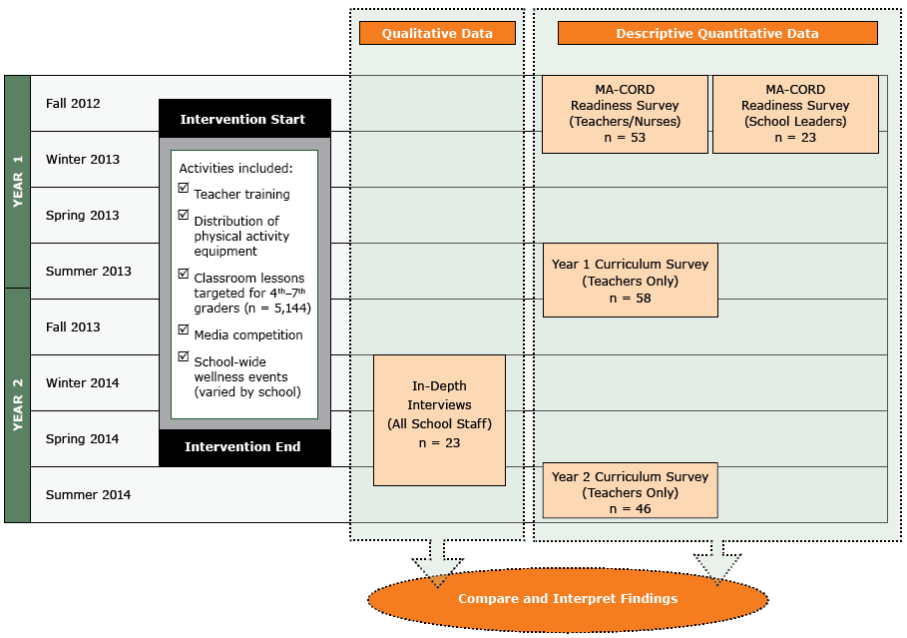
MA-CORD school sector implementation data used in a convergent parallel mixed methods design. The MA-CORD intervention occurred over a 2-year period and was evaluated using both quantitative and qualitative measures.

We employed a convergent, parallel mixed-methods design (11) to examine facilitators and barriers to implementing MA-CORD. Informed by the taxonomy of Proctor et al of outcomes for implementation research (9), outcomes included were acceptability, adoption, appropriateness, feasibility, implementation fidelity, perceived implementation cost, reach, and sustainability. Throughout the intervention we collected data from school staff members using both qualitative methods (ie, in-depth interviews) and quantitative methods (eg, cross-sectional surveys) to assess these outcomes (Figure 1). Our design was ideally suited for process evaluation because interview findings provided context for outcomes not easily explained through survey data alone.

For both in-depth interviews and readiness surveys we used a convenience sample of school leaders (eg, principals, community coordinators, wellness champions) and staff members (eg, teachers, school nurses) in MA-CORD schools in Community 1 (n = 6) and Community 2 (n = 22). End-of-year curriculum surveys were collected from eligible teachers. The number of eligible teachers varied slightly by year in Community 1 (n = 7 in year 1; n = 6 in year 2) and Community 2 (n = 117 in year 1; n = 122 in year 2). Interviewees from each community were principals and superintendents (n = 5), wellness champions and school nurses (n = 11), and teachers eligible to offer the curricula (n = 7).

Two anonymous surveys were administered at baseline to assess stakeholder readiness for implementing MA-CORD (Figure 1). In addition, 2 anonymous surveys were administered to teachers at the end of each academic year to assess the delivery of the MA-CORD intervention. These surveys were administered online via Qualtrics Insight (Qualtrics) or pen-to-paper (Appendix A, Appendix B). In-depth interviews were conducted by telephone with school leaders and staff members in year 2 to assess implementation of MA-CORD activities. Study procedures were approved by the human subjects committees of the Massachusetts Department of Public Health, Harvard T.H. Chan School of Public Health, Massachusetts General Hospital, and Harvard Pilgrim Health Care Institute in June 2012 (#331765).

### Measures

**Readiness surveys**. Two measures of organizational readiness for change were used to measure program acceptability. The first, provided to school leaders, contained items adapted from an existing tool (12) and assessed school and district readiness for adoption and leadership support for MA-CORD. The second survey given to school staff (eg, teachers, nurses) contained items adapted from an existing readiness-for-change scale for employees within an organization (13,14) to assess staff engagement and support for MA-CORD.

**Curriculum surveys**. Curriculum surveys collected at the end of years 1 and 2 assessed appropriateness (eg, lessons perceived as positive addition to curriculum), feasibility, perceived implementation cost (eg, perceived competence to teach curriculum, perceived effort to obtain materials to complete lessons), implementation fidelity (eg, proportion of MA-CORD lessons taught), and sustainability (eg, plans to continue offering the lessons in the following year).

**In-depth interviews**. Using semi-structured interview guides, participants were asked about appropriateness of MA-CORD, barriers and facilitators to adoption, implementation fidelity, perceived intervention cost, and changes in activities over time. To examine sustainability of MA-CORD activities, participants were also asked about intervention reach based on links to activities in their school and community.

**Internal records**. For each community, we obtained a census roll of superintendents, principals, school nurses, school coordinators, wellness champions, and eligible teachers. These records were updated regularly on the basis of reports from internal research group meetings (eg, staff layoffs, medical leave) or delays in intervention activities (eg, snow days). Sign-in sheets indicated the number of teachers who completed the MA-CORD curriculum training.

### Data analysis

We used SAS 9.3 (SAS Institute) to generate descriptive statistics including means, standard deviations, and frequencies for survey and internal record data. Interviews were digitally recorded, transcribed verbatim, and analyzed using NVivo 10 (QSR International). A coding scheme was developed based on a conceptual framework (9) and piloted with 5 transcripts among 3 coders to ensure internal consistency (Appendix C). Transcripts were double coded using the constant comparative method (15) to identify emergent themes, and discrepancies were discussed through peer review to clarify coded passages and resulting themes. Finalized themes within implementation outcome categories were coded and summarized within and across both MA-CORD communities (Appendix D). Qualitative and quantitative data were triangulated across outcomes to identify factors that influenced implementation.

## Results

Table 1 summarizes characteristics of communities, schools, students and staff. Quantitative and qualitative measures were used to assess outcomes based on the taxonomy for implementation research outcomes of Proctor et al (9) (Table 2). MA-CORD implementation barriers and facilitators were assessed during year 2 using in-depth interviews and summarized based on implementation outcomes (Table 3).

**Table 1:** Characteristics of Communities, Schools, Students, and Staff Members Participating in the MA-CORD Intervention, Massachusetts, 2012–2014

Characteristic	Community 1	Community 2
**Community**		
**Population total (** 30 **), n **	40,318	95,072
**Race/ethnicity (** 30 **), % **		
White	68.2	67.9
Hispanic	21.6	16.7
African American	5.1	6.4
Multi-race, Non-Hispanic	3.7	5.7
Asian	3.6	0.9
**Average per capita income **(30)**, $**	22,620	21,056
**Persons below poverty level **(30)**, %**	20.6	23.5
**School**		
**Schools eligible to participate in MA-CORD^a^ , n**	6	22
Elementary schools	4	19
Middle schools	3	3
**Health education staff**		
Schools with nurses, n	6	25
Schools with a health education teacher, n (% of schools)	6 (100.0)	3 (13.6)
**District-wide staff retention rates, n (% of schools)**		
Superintendent	1 (100.0)	0 (0.0)
Principals	7 (87.5)	19 (79.2)
Teachers	315 (92.9)	777 (90.0)
**Teacher**		
**Total eligible to teach MA-CORD curricula, n**		
Year 1	7	117
Year 2	6	122
**Female, % of eligible teachers **(31)	81.3	81.4
**Race/ethnicity, % of eligible teachers **(31)		
White	90.5	90.7
Hispanic	6.8	2.5
African American	2.0	5.7
Multi-race, Non-Hispanic	0.2	0.6
Asian	0.5	1.3
**Student**		
**Total eligible to receive MA-CORD curricula^b^ (** 31 **), n **	1486	3658
**Race/ethnicity, % of students (** 31 **)**		
White	38.2	49.2
Hispanic	46.6	31.1
African American	5.8	11.7
Multi-race, Non-Hispanic	5.7	6.1
Asian	5.5	0.8
**Low-income^c^ (** 31 **)**	76.9	73.4
**Engagement in Process Evaluation**		
**Surveys of intervention readiness^d^**		
Leaders^e^, n	5	18
Teachers or nurses, n	4	49
**Qualitative interviews, n**		
Schools represented in qualitative interviews, n (% of schools)^f^	5 (83.3)	11 (50.0)
Leaders^e^, n	4	2
Teachers or nurses, n	7	10
**Year-end teacher curriculum surveys, n (% of teachers)**		
Year 1	7 (100)	51 (43.6)
Year 2	5 (83.0)	41 (33.6)

Abbreviation: MA-CORD: Massachusetts Childhood Obesity Research Demonstration Project.

a Community 1 consisted of 6 schools, but 1 school served kindergarten through eighth-grade students and was counted as both an elementary and a middle school.

b Students enrolled in fourth, fifth, sixth, and seventh grade were eligible to receive the curricula used in MA-CORD.

c Defined as being eligible for either free or reduced price lunch, transitional aid to families, or the Supplemental Nutrition Assistance Program based on family household income.

d Intervention readiness surveys were distributed to MA-CORD school leaders and staff members (Table 2); participants were not identified by school.

e School principals, superintendents, intervention coordinators, and MA-CORD wellness champions.

f In-depth qualitative interviews conducted during year 1 of the intervention with school leaders (superintendent, principals, wellness champions), teachers, and nurses.

**Table 2:** Outcomes of an Implementation Assessment of MA-CORD School-Based Intervention^a^, Massachusetts, 2012–2014

Measures	Community 1	Community 2
**Acceptability^b^**		
**Beliefs of school leaders^c^^,^ d, mean (standard deviation)**		
Commitment to prevent or reduce childhood obesity in the community	4.9 (0.2)	4.7 (0.2)
Compatibility of program with organization’s approach	4.2 (0.8)	4.3 (0.5)
Timing of implementation was good	4.3 (0.7)	4.0 (0.6)
Intervention will distract from other organizational priorities	2.4 (0.7)	1.7 (0.5)
**Beliefs of school staff members^d^^,^ e, mean (standard deviation)**		
Commitment of staff to implementation	4.2 (0.5)	3.8 (0.9)
Motivation of staff for implementation	4.3 (0.5)	3.6 (0.8)
Confidence of staff to implement tasks smoothly	4.0 (0.8)	3.6 **(**0.9)
Confidence of staff to handle implementation challenges	4.3 (0.5)	3.6 (0.8)
Confidence of staff members that organization can support them during transition to intervention	4.3 (0.5)	3.6 (0.8)
**Adoption^f^**		
**Teacher adoption of MA-CORD lessons, n (% of teachers)**		
Eligible teachers completed MA-CORD curriculum training in year 1^g^	7 (100.0)	84 (71.8)
Taught any MA-CORD lessons in year 1^h^	7 (100.0)	28 (59.6)
Taught any MA-CORD lessons in year 2^i^	5 (100.0)	39 (75.0)
**Appropriateness^j^**		
“Lessons I taught were a positive addition to my curriculum” (Agree or strongly agree)^i^	7 (100.0)	28 (100.0)
**Feasibility^k^/Perceived Implementation Cost^l^**		
**Beliefs of MA-CORD eligible teachers, n (%)**		
“I felt competent to teach the content” (agree or strongly agree)^i^	6 (85.7)	25 (56.8)
“Overall, the effort required to obtain needed materials not provided [by MiM Kids] was acceptable”^i^	4 (80.0)	29 (90.6)
**Beliefs of school leaders^c^^,^ d, mean (standard deviation)**		
Organization has resources necessary for implementation	3.5 (0.8)	3.8 (0.8)
Organization can manage risks associated with implementation	3.7 (1.0)	3.7 (0.5)
**Implementation Fidelity^m^**		
Lessons taught from MA-CORD curriculum in year 1 (mean, SD)^n^	5.8 (2.7)	3.6 (2.5)
Lessons taught from MA-CORD curriculum in year 2 (mean, SD)^n^	5.2 (3.0)	4.5 (2.8)
**Reach^o^**		
Estimated number of students who received MA-CORD curriculum^h^ (31)	1,486	2,262
**Sustainability^p^**		
**Teachers sustaining MA-CORD curriculum, n (%)**		
Plan to teach curriculum after year 1 (yes vs no/undecided)^h^	7 (100.0)	40 (83.3)
Plan to teach curriculum after year 2 (yes vs no/undecided)^i^	5 (100.0)	29 (76.3)

Abbreviations: MA-CORD, Massachusetts Childhood Obesity Research Demonstration Project; MiM KIDS, Mass in Motion KIDS intervention.

a The community-level name for the intervention that was part of the larger MA-CORD project was MiM KIDS.

b Acceptability is the initial perception of the intervention’s fit.

c Data obtained from survey of leaders in the school sector (administrators, principals, school wellness champions) using an adapted version of the Adoption Decision Questionnaire: Community 1 (n = 5), Community 2 (n = 18).

d Response options ranged from 1 (strongly disagree) to 5 (strongly agree).

e Data obtained from survey of staff members in the school sector (teachers, school nurses) using an adapted version of the Organizational Readiness for Change Questionnaire: Community 1 (n=4), Community 2 (n = 49).

f Adoption in initial participation.

g Based on sign-in sheets and internal records.

h Data obtained from year 1 curriculum survey of staff members eligible to teach MA-CORD curriculum: Community 1 (n = 7), Community 2 (n = 51).

i Data obtained from year 2 curriculum survey of staff members eligible to teach MA-CORD curriculum: Community 1 (n = 5), Community 2 (n = 41).

j Appropriateness is the perception of MiM Kids as being good for teachers/children

k Feasibility is the actual fit/compatibility of conducting MiM Kids activities in a school setting.

l Perceived implementation cost refers to the resources required to conduct activities (eg, financial, time, parent support).

m Implementation fidelity is the quantity and quality of MiM Kids activities conducted.

n Compared with goal of 6 MA-CORD lessons taught per year.

o Reach is the impact of MiM Kids on students, parents, staff, and community.

p Sustainability is the continuation/institutionalization of MiM Kids activities.

**Table 3:** Barriers and Facilitators to Implementation of the MA-CORD School-Based Intervention Based on In-Depth Interviews of School Administrators, Teachers, and Nurses (n = 23)^a^, Massachusetts, 2013–2014

Implementation Outcome Constructs	Facilitators^b^	Barriers^b^
**Acceptability^c^**	Principal is a champion for health activities	Pressure of standardized testing or academic demands in district
	Existing wellness initiatives and policies (C1)	New superintendent and administrative turnover (C2)
	School nurses and health education teachers found the project fit well within their work tasks	
**Adoption^d^**	Rapport between wellness champions and the staff	Weather interrupting trainings (C2)
		Lack of time for teachers to attend trainings
		Teachers not informed about intervention (C2)
**Appropriateness^e^**	Training and curricula were well-received	Concerns about messages that children do not have control over (eg, safe outdoor play, sleep environments)
	Message appropriate for students	
	Teachers liked being part of a larger movement across schools	
**Feasibility^f^/implementation fidelity^g^**	A champion at the school who maintains enthusiasm	Lack of time for teachers to teach lessons
	Using students to engage other students	Competing priorities with other schoolwide campaigns
	Technical assistance to change policies in the school	Principal and teacher turnover (C2)
**Perceived implementation cost^h^**	Providing physical activity equipment to schools (C2)	Inadequate printing resources to provide materials for conducting lessons
**Reach^i^**	School-wide integration of messaging	Limited collaboration between some sectors
	Linkages with other school health priorities	
	Media coverage	
	Children bringing messages home from school	
**Sustainability^j^**	Health education teachers implementing curriculum	Staff turnover
	Enjoyable activities that are adopted long-term	Lack of ongoing leadership
	Intervention involvement acknowledged in teacher evaluations	

Abbreviations: C1, Community 1; C2, Community 2; MA-CORD Project, Massachusetts Childhood Obesity Research Demonstration Study.

a Based on sample of 11 school staff members in Community 1 and 12 school staff members in Community 2.

b Themes reported in both communities unless otherwise specified.

c Acceptability: Initial perception of intervention fit.

d Adoption: Initial participation.

e Appropriateness: Perception of Mass in Motion [MiM] Kids being good for teachers/children (MiM KIDS was the community-level name for the intervention that was part of the larger MA-CORD project).

f Feasibility: Actual fit/compatibility of conducting MiM Kids activities in school setting.

g Implementation Fidelity: Quantity and quality of MiM Kids activities conducted.

h Perceived implementation cost: Resources required to conduct activities.

i Reach: Impact of MiM Kids on students, parents, staff, and community.

j Sustainability: Continuation/institutionalization of MiM Kids activities.

**Acceptability**. Before the intervention, leaders in both districts reported high levels of support for MA-CORD (Table 2 **)**. Among school staff members, scores for organizational commitment, motivation, and confidence in their school’s ability to support MA-CORD were lower in Community 2 than Community 1. In interviews, staff members in Community 2 discussed concerns about changing administrative priorities and focusing on standardized testing, which competed with outside activities. Acceptability facilitators were preexisting wellness activities related to nutrition and physical activity, parental involvement, and strong principal support.

**Adoption**. Teachers in both communities participated in MA-CORD curriculum training (C1:100%; C2:72%) and in a curriculum survey in year 1, which assessed initial adoption (C1:100%; C2:44%). Most teachers reported teaching at least one lesson during both year 1 (C1:100%, C2:60%) and year 2 (C1:100%; C2:75%) (Table 2). During interviews, participants from Community 2 described difficulty coordinating afterschool schedules of teachers for training sessions. Teachers in both communities described motivated wellness champions as a driving force behind adoption of MA-CORD lesson plans.

**Appropriateness**. In interviews, teachers and staff members in both communities reported that MA-CORD training and curricula were appropriate for their students and teaching priorities. In curriculum surveys, teachers in both communities unanimously agreed (n = 35, 100%) that the lessons were a positive addition to their curriculum.

**Feasibility**. Although teachers in both communities reported being able to obtain necessary lesson materials (>80%), fewer teachers in Community 2 reported feeling competent to teach the content (Community 2, 57% vs Community 1, 86%). In interviews, participants across both communities identified competing priorities for teachers’ time as barriers to administering classroom lessons. Standardized tests, statewide campaigns (anti-bullying curriculum), and general burnout were cited as barriers to the staff teaching lessons on wellness or being involved in wellness activities.

**Figure 2:**
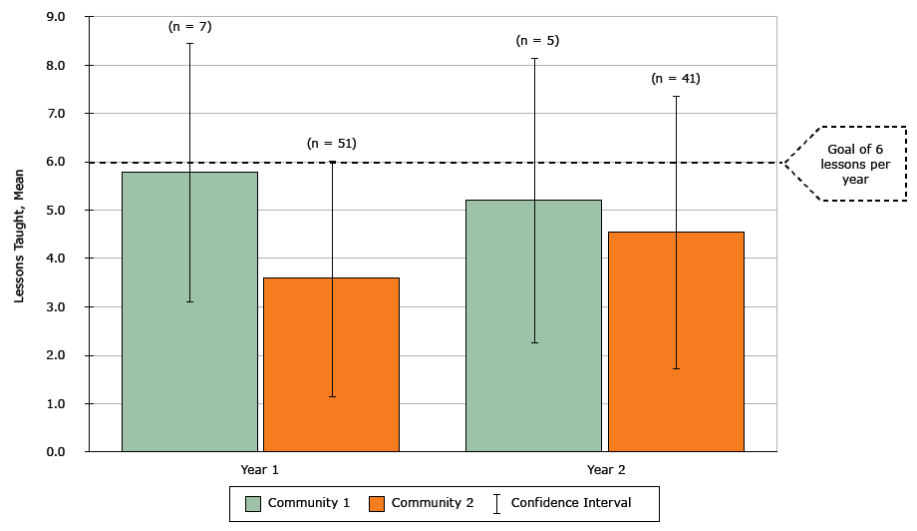
MA-CORD Implementation Fidelity: Curriculum lessons taught by 4th, 5th, 6th, and 7th grade school teachers, Massachusetts, 2012–2014. Using end-of-year surveys, teachers reported the number of lessons taught from the MA-CORD curricula, which were adapted from “Eat Well and Keep Moving” and “Planet Health” (Appendix A). **Table:** 

**Implementation fidelity**. In year 1, teachers in Community 1 nearly met the teaching goal of 6 MA-CORD lessons per year (mean, 5.8: standard deviation [SD], 2.7); Community 2 reported fewer lessons (mean, 3.6; SD, 2.5) (Figure 2). In year 2, mean lessons taught dropped slightly for Community 1 and increased for Community 2. In Community 2, administrative changes, including a new superintendent, principal turnover, and district-wide teacher layoffs, were described in interviews as barriers to implementation fidelity.

Year	Community 1	Community 2
	Mean (Standard Deviation)	
1	5.8 (2.7)	3.6 (2.5)
2	5.2 (3.0)	4.5 (2.8)

**Perceived implementation cost**. In surveys, school leaders in both communities were neutral or agreed that their schools had resources to support MA-CORD and could manage risks associated with implementing the intervention. In interviews, leaders and staff members in both communities reported satisfaction with the availability of supplies and resources needed to implement activities. Community 2 staff members reported receiving physical activity play equipment as a major benefit of MA-CORD participation.

**Reach**. On the basis of the number of 4th through 7th grade students eligible to receive the intervention; (Community1: 1,486; Community 2: 3,658) (Table 1) and the percentage of eligible teachers who completed trainings (Community 1, 100%; Community 2, 72%) (Table 2), we estimate that 1,486 students in Community 1 (100%) and 2,626 students in Community 2 (72%) were reached by the intervention. In interviews, leaders and staffs in both communities reported classroom activities effectively tied into larger school and city-wide campaigns, thus increasing student and family awareness.

**Sustainability**. In end-of-year curriculum surveys in year 2, most teachers in Community 1 (100%, n = 5) and Community 2 (76%, n = 29) reportedly planned to continue teaching MA-CORD lessons. In interviews, staff members described health teachers as strong implementers of the curriculum. One principal made MA-CORD activities part of teachers’ professional evaluation, ensuring MA-CORD lessons would be sustained through supervisory accountability. Barriers to long-term sustainability were teacher turnover, lack of ongoing leadership from principals, or lack of active wellness champions.

## Discussion

Our study describes barriers and facilitators to implementing a school-based obesity intervention in 2 low-income communities. MA-CORD was adopted at a rate comparable to similar classroom-based lifestyle interventions (16–18) in districts facing competing priorities. Understanding factors facilitating implementation is necessary to develop targeted technical assistance and resources for successful implementation. Our findings provide insight into benefits of pre-intervention assessment of staff readiness and selection of ideal teachers and curricula to ensure activities are integrated and sustained in schools. Our study yielded 4 key lessons learned:

**Lesson 1: Assess organizational readiness of all staff members.** Strong leadership support for MA-CORD existed in both communities at baseline, but implementers (ie, teachers, nurses) in Community 2 reported lower perceived readiness to implement MA-CORD than did implementers in Community 1. In fact, proportionally fewer teachers in Community 2 engaged in training, taught lessons, completed curriculum surveys, or planned to sustain lessons post-intervention. These teachers described administrative shifts and staff turnover (45% of schools in Community 2 received new principals), in contrast with administratively stable Community 1, which also had a history of parent involvement and wellness activities before MA-CORD.

Health education teachers administered lessons in Community 1, whereas a mix of health education teachers and classroom teachers in Community 2 administered them. In low-resourced communities with few health education teachers, additional strategies to identify motivated teachers or parents could be beneficial. Lack of parental involvement is reported as a barrier to implementation in school-based obesity prevention projects serving low-income children (19,20). Interviewees suggested parents could support teachers delivering MA-CORD lessons by bringing healthy snacks to taste-test or by planning school wellness events. In future projects, school leaders should consider collaboratively addressing barriers to implementation by increasing parental involvement before launching intervention activities.

**Lesson 2: Identify and support passionate wellness champions.** Using school wellness champions was one of the strongest reported facilitators of MA-CORD implementation, consistent with previous research indicating the use of outside staff to implement an intervention significantly reduced its likelihood of being sustained (21). We found that champions who were health education teachers or nurses reported the highest satisfaction with their role because it fit well with their job description. In Community 2, busy principals and classroom teachers served as wellness champions, but some colleagues reported waning support from them because of shifting administrative priorities over time.

Although some schools may not have health education teachers or nurses who can take on additional roles, investigators may increase engagement and buy-in from champions by using strategies adapted from workplace wellness programs: ongoing training, recognition, and incentive programs linked with key intervention outcomes (22,23). Wellness champions who efficiently train and motivate busy teachers to adopt new classroom activities play a critical role in implementation success. These champions are also likely to support overall district and school-level wellness policy implementation.

**Lesson 3: Build on existing curricula combined with incentives**. Tailored messaging and print materials are valuable contributors to successful obesity-related intervention outcomes in school-based settings (24). In our study, teachers consistently conveyed satisfaction with the lesson plans and print materials adapted from existing interventions. For example, one *Eat Well and Keep Moving* lesson titled “Sugar Water: Think about Your Drink,” contained activities crossing various core curricula (eg, multiplication to find grams of sugar in soda, interpreting a soda can label). Obesity prevention lessons that fulfill multiple core classroom subjects support adoption and sustainability of intervention activities in schools (18). Curriculum delivery was maximized by incentivizing aspects of program participation with grant funding. Teachers were compensated for attending MA-CORD training sessions after school or they attended sessions during the school day, which probably contributed to greater than 70% teacher participation in both communities. As an additional incentive, some schools received play equipment such as balls and hula hoops, which promoted active indoor play during winter months and supported the intervention’s physical activity goal.

**Lesson 4: Sustainability is maximized through ongoing training and institutional adoption**. Teachers who continued to teach MA-CORD lessons beyond year 1 of the intervention described having a wellness champion who offered ongoing support through formal and informal training. Both in our study and elsewhere, staff turnover is a barrier to intervention sustainability in schools, because repeated training is expensive and difficult to coordinate across campuses (25–27). However, we identified sustainable strategies, which included incorporating the curricula into lesson plans that continued year-to-year (eg, math lessons, writing), acknowledging MA-CORD activities in performance evaluations, and schoolwide policies supporting messages taught during lessons (eg, no sugary drinks on campus). Additionally, online training modules are being considered as a low-cost way to train a school’s staff on health topics (28) and could be a way to overcome issues related to staff turnover. One study found no significant difference in adoption of an after-school nutrition and physical activity intervention when the staff were trained online versus face-to-face (29).

As in other process analyses, our study’s findings rely on self-report from a convenience sample (17). In one community, nearly half of eligible teachers did not complete follow-up curriculum surveys, reflecting possible unmeasured levels of implementation in nonparticipating schools. Because student-level data were not collected because of privacy restrictions, we based our estimate of reach on the number of eligible students and percentage of eligible teachers who attended MA-CORD trainings. Although small sample sizes limited our ability to generalize beyond our population, using mixed methods offered detailed context, which may be useful for others working to implement similar programs in resource-poor schools. Because long-term follow-up data beyond the intervention period were not available, we could not assess the intervention’s long-term sustainability.

To improve child health and maximize limited resources, there remains a need for continued collection and publication of both quantitative and qualitative process evaluation data describing school-based obesity prevention interventions. Sharing null findings, barriers, and implementation failure is critical to refining and promoting best practices in implementation to identify strategies to encourage sustainable changes in schools.

## Appendix A – Questionnaires Used in Process Evaluation of School Intervention

This file is available for download as a Microsoft Word file.

## Appendix B – Interview Guides

This file is available for download as a Microsoft Word file.

## Appendix C – Interview Coding Scheme

This file is available for download as a Microsoft Word file.

## Appendix D – Key Illustrative Quotes Obtained From Qualitative Interviews of School Staff Members Participating in MA-CORD in Massachusetts, 2012–2013 (n = 23)

This file is available for download as a Microsoft Word file.
