# Supplementary material for: Using School Staff Members to Implement a Childhood Obesity Prevention Intervention in Low-Income School Districts: the Massachusetts Childhood Obesity Research Demonstration (MA-CORD Project), 2012–2014
Source: Prev Chronic Dis. 2017 Jan 12;14:E03. doi: 10.5888/pcd14.160381 (PMC5234440; doi:10.5888/pcd14.160381)
Supplement: Supplementary file 1 [file 16_0381AppendixA.docx]

**Appendix A – Questionnaires Used in Process Evaluation of School Intervention**

**EAT WELL AND KEEP MOVING TEACHER SURVEY**

This survey is part of a research study being undertaken by the Massachusetts Department of Public Health, in collaboration with the Harvard School of Public Health, with the cooperation of the school district. Research staff from the Harvard School of Public Health will be collecting the survey results. You are invited to take part in this survey because we want to understand your experience as a teacher delivering classroom lessons related to the *Mass in Motion Kids* project in your community. Participation in this survey is voluntary. If you choose to take part, you may change your mind and stop the survey at any time.   We are interested in which lessons you taught and your experiences teaching them. This survey should take approximately 10 minutes to complete. Information from this survey will help us understand which lessons students received, whether there were any barriers in delivering the lessons and teacher perceptions of how the lessons might have influenced students. **Thank you for your participation!**

1. Date (mm/dd/yy )____________________
2. I am a: Classroom teacher Health teacher PE teacher Not a Teacher Other ________________
3. ***If you did not teach any Eat Well and Keep Moving lessons since_______, CHECK HERE* and go to question 14.**
4. Identify the lessons you taught since ________. In the Subject Area column, please write the subject area during which you taught the lesson. In the grade columns, please check if you taught the corresponding lesson to the grade. Use the “other” column for multi-grade classes.

| **Subject Area** | **Lesson** | **4^th^** | **5th** | **Other** |
| --- | --- | --- | --- | --- |
|  | Lesson 1: Healthy Living |  |  |  |
|  | Lesson 2: Carb Smart |  |  |  |
|  | Lesson 3: The Safe Workout – An Introduction |  |  |  |
|  | Lesson 7: Sugar Water: Think about Your Drink |  |  |  |
|  | Lesson 9: Prime Time Smartness |  |  |  |
|  | Lesson 10: Chain 5 |  |  |  |
|  | Lesson 14: Healthy Living, Healthy Eating |  |  |  |
|  | Lesson 16: The Safe Workout - A Review |  |  |  |
|  | Lesson 18: Beverage Buzz |  |  |  |
|  | Lesson 19: Snack Decisions |  |  |  |
|  | Lesson 21: Freeze My TV |  |  |  |
|  | Lesson 22: Menu Monitoring |  |  |  |

**For the questions below, check the box that best fits your opinion:**

1. Overall, the *Eat Well and Keep Moving* lessons I taught were a positive addition to my curriculum.

Strongly Disagree Disagree Agree Strongly Agree

1. I was able to adapt the lessons I taught to meet my needs.

Strongly Disagree Disagree Agree Strongly Agree

1. I felt competent to teach the content.

Strongly Disagree Disagree Agree Strongly Agree

1. Overall, the effort required to obtain needed materials not provided by *Eat Well and Keep Moving* was acceptable.

Strongly Disagree Disagree Agree Strongly Agree

9. Overall, I think *Eat Well and Keep Moving* has had a *…*

positive effect no effect negative effect on my students

positive effect no effect negative effect on my teaching

positive effect no effect negative effect on my ability to connect with my students

10. Did you attend an *Eat Well and Keep Moving* workshop during the ______ school year? Yes No

11. If you answered “no” in the previous question, which reply best describes why you did not attend a workshop:

Scheduling conflicts Already had enough training Felt workshop did not meet my needs Other__________________

12. Do you plan to teach *Eat Well and Keep Moving* next year? Yes No Undecided

13. Were any obstacles encountered during *Eat Well and Keep Moving* implementation? Yes No

If you answered “yes”, please describe:

14. If you have NOT used *Eat Well and Keep Moving* yet, why not?

Not enough time The curriculum doesn’t seem grade appropriate.

Not enough administrative support The content is too difficult to teach.

I/we didn’t like the lessons. My organization doesn’t need health curriculum now.

I/we don’t know how to use the curriculum.

Other – *Please specify*

15. List any special events or programs you coordinated (or worked on) this year related to physical activity and nutrition for students or staff. Attach extra sheets if necessary. Please attach fliers or other promotional material if available.

**PLANET HEALTH TEACHER SURVEY**

This survey is part of a research study being undertaken by the Massachusetts Department of Public Health, in collaboration with the Harvard School of Public Health, with the cooperation of the school district. Research staff from the Harvard School of Public Health will be collecting the survey results. You are invited to take part in this survey because we want to understand your experience as a teacher delivering classroom lessons related to the *Mass in Motion Kids* project in your community. Participation in this survey is voluntary. If you choose to take part, you may change your mind and stop the survey at any time.   We are interested in which lessons you taught and your experiences teaching them. This survey should take approximately 10 minutes to complete. Information from this survey will help us understand which lessons students received, whether there were any barriers in delivering the lessons and teacher perceptions of how the lessons might have influenced students.

1. Date (mm/dd/yy )____________________
2. What subject(s) do you teach? Health/CFS Language Arts Math P.E. Science Social Studies Not a Teacher Other _______________
3. ***If you did not teach any Planet Health lessons since_______, CHECK HERE* and proceed to question 14.**
4. Identify the lessons you taught since ________. In the Subject Area column, write the subject area during which you taught the lesson. In the Grade columns, check if you taught the corresponding lesson to the grade. Use the “other” column for multi-grade classes.

| **Subject Area** | **Lesson** | **6^th^** | **7th** | **Other** |
| --- | --- | --- | --- | --- |
|  | Lesson 1 (Intro lesson): Do You Make Space for Fitness and Nutrition? |  |  |  |
|  | Lesson 2 (Intro lesson): Power Down- Charting Screen Time |  |  |  |
|  | Lesson 19: (Science) Passing the Sugar |  |  |  |
|  | Lesson 8 (Language Arts): Go for the Goal |  |  |  |
|  | Lesson 5: The Language of Food (Language Arts) |  |  |  |
|  | Lesson 9: (Language Arts) Lifetime Physical Activities: Research One, Describe One, Try one! |  |  |  |
|  | Lesson 6: (Language Arts) Keep It Local |  |  |  |

**For the questions below, check the box that best fits your opinion:**

1. Overall, the *Planet Health* lessons I taught were a positive addition to my curriculum.

Strongly Disagree Disagree Agree Strongly Agree

1. I was able to adapt the lessons I taught to meet my needs.

Strongly Disagree Disagree Agree Strongly Agree

1. I felt competent to teach the content.

Strongly Disagree Disagree Agree Strongly Agree

1. Overall, the effort required to obtain needed materials not provided by *Planet Health* was acceptable.

Strongly Disagree Disagree Agree Strongly Agree

9. Overall, I think *Planet Health* has had a *…*

positive effect no effect negative effect on my students

positive effect no effect negative effect on my teaching

positive effect no effect negative effect on my ability to connect with my students

10. Did you attend a *Planet Health* workshop during the ______ school year? Yes No

11. If you answered “no” in the previous question, which reply best describes why you did not attend a workshop:

Scheduling conflicts Already had enough training Felt workshop did not meet my needs Other__________________

12. Do you plan to teach *Planet Health* next year? Yes No Undecided

13. Were any obstacles encountered during *Planet Health* implementation? Yes No

If you answered “yes”, please describe:

14. If you have NOT used *Planet Health* yet, why not?

Not enough time The curriculum doesn’t seem grade appropriate.

Not enough administrative support The content is too difficult to teach.

I/we didn’t like the lessons. My organization doesn’t need health curriculum now.

I/we don’t know how to use the curriculum.

Other – *Please specify*

15. List any special events or programs you coordinated (or worked on) this year related to physical activity and nutrition for students or staff. Attach extra sheets if necessary. Please attach fliers or other promotional material if available.

**Thank you for your participation!**
