# Supplementary material for: Using School Staff Members to Implement a Childhood Obesity Prevention Intervention in Low-Income School Districts: the Massachusetts Childhood Obesity Research Demonstration (MA-CORD Project), 2012–2014
Source: Prev Chronic Dis. 2017 Jan 12;14:E03. doi: 10.5888/pcd14.160381 (PMC5234440; doi:10.5888/pcd14.160381)
Supplement: Supplementary file 2 [file 16_0381AppendixB.docx]

**Appendix B – Interview Guides**

**Interview Guides – MA-CORD School Implementers (teachers)**

| **Construct #1: Warm‐up/ Baseline** | 1. Can you please briefly describe your role in your (organization) including your job title and the general things you are responsible for? |
| --- | --- |
| **Construct #2: Organizational and individual role in MiM Kids** | 1. From your perspective, what is MiM Kids? 2. What is your organization’s role in implementing MiM Kids? 3. What is YOUR role in MiM Kids? 4. What specific things have you done as part of MiM Kids? |
| **Construct 3: Institutional Fit**  **(No Child Care Mentors)** | 1. Does MiM Kids fit with your organization’s priorities?    1. Do you feel it is a high, medium, or low priority for your organization?    2. What gives you that impression? 2. Were any competing priorities voiced by staff? 3. Does MiM Kids fit with your current work tasks and job description?    1. **If not:** Why not? Can you please explain that a little bit? 4. How much of your time or effort is required for MiM Kids activities (**Probe for:** per day or week)?    1. Has the amount of time or effort changed over time? **If yes:** In what ways? 5. Have your responsibilities for MiM Kids changed over the past year? **If yes:** How? 6. Do you feel that your role and work in MiM Kids is valued and recognized?   **If yes OR no:** Can you tell me more about why you feel that way? **(Probe for:** How their job is recognized/valued; specific people that they feel do/don’t value their work; other examples.   1. How engaged and/or supportive are staff members (e.g. teachers, principals, school nurses, etc.) of MiM Kids? **Probe for:** Examples. |
| **Construct #4: Training and resources** | 1. I know that school nurses didn't receive any specific training to prepare them for their roles in MiM Kids, but are there any trainings that would have helped you to prepare you for this role? |
| **Construct #5: Successes and Barriers; time commitment** | 1. Thinking back over your experiences with MiM Kids over the past year, what do you think has been working well?    1. Do you have any successes to share? 2. What problems or challenges (if any) have you, or the staff implementing MiM Kids, experienced? 3. Are there any ways you feel that MiM Kids components could be redesigned to improve them and/or make them more feasible for you or your colleagues to successfully implement? **Probe for:** How? |
| **Construct #6: Parent engagement** | 1. Are families concerned and aware about childhood obesity? 2. To what extent are parents engaged in combating or preventing childhood obesity? 3. How, if at all, has parent’s awareness of and/or engagement in this issue changed since MiM Kids was launched? 4. What do you think is necessary to increase parent engagement and awareness of childhood obesity prevention? |
| **Construct #7: Changes over time** | 1. Have there been any major changes in your organization since MiM Kids started? **Probe for** changes in staffing, and positive and negative changes.    1. **If yes:** What changes have been made?    2. What implications (if any) have these changes had for the implementation of MiM Kids? 2. Tell me about other initiatives your organization has implemented in the past year to prevent and control childhood obesity? 3. Were these initiatives launched before or after MiM Kids was implemented? 4. At your organization: Do your initiatives dealing with childhood obesity complement the MiM Kids program or is there overlap? |
| **Construct #8: Linkage** | 1. Have you noticed any connection between MiM Kids activities within your organization and obesity prevention efforts within the broader community? 2. To your knowledge, have children who are overweight or obese been referred to other obesity prevention programs in your community (e.g. Healthy Weight Clinic, after school programs)? 3. As part of MiM Kids, do you interact with other sectors (e.g. school system, health clinics, after school, child care, parks and recreation) in the community?   **If yes:**   - 1. Which sectors do you interact with?   2. What challenges do you encounter when communicating with the other sectors? **Probe for:** What works well, what doesn’t? Why or why not? |
| **Construct #9: Media Competition**  **(School Personnel Only)** | 1. Have you heard of the MiM Kids School Media Competition where kids in schools and after-school programs created video, song, or rap or artwork (like a poster)?   **If no:** Skip to Q 28.  **If yes:**   - 1. Was this competition implemented at your school?   **If yes:**   - 1. What was the general impression of the media competition in your school?   2. Did you get feedback about the competition from the broader community? **If yes:** What kind?   3. What are your thoughts about how it was implemented in your school?   4. What benefits do you think the media competition had for schools and after-school programs in general? What about benefits for the students?   5. How well equipped did you feel to help students and schools participate in the competition?   6. What changes, if any, would you recommend in the process?   **If no:**   - 1. What were some reasons why it was not implemented at your school?   2. What was the general impression of the media in the broader community?   3. What benefits do you think the media competition had for schools and after-school programs in general? What about benefits for the students?   4. What changes, if any, would you recommend in the process? |
| **Construct #10: Closing** | 1. If you were giving the choice to be a part of MiM Kids again, would you choose to?   **If yes:** Why?  **If no:** Why not?   1. Is there anything I haven’t asked about MiM Kids that you think is important for me to know? |

**Interview Guide – School Leaders**

| **Construct #1: Warm‐up/ Baseline** | 1. Can you please briefly describe your role in your organization including your job title and the general things you are responsible for? |
| --- | --- |
| **Construct #2: Organizational and individual role in MiM Kids** | 1. From your perspective, what is MiM Kids? 2. What is your organization’s role in implementing MiM Kids? 3. What is YOUR role in MiM Kids? 4. What specific things have you done as part of MiM Kids? 5. In what ways do you communicate information about MiM Kids to staff?   **Probe for:** How regularly? With whom? |
| **Construct 3: Institutional Fit** | 1. Does MiM Kids fit with your organization’s priorities?    1. Do you feel it is a high, medium, or low priority for your organization?    2. What gives you that impression?    3. Were any competing priorities or concerns voiced by staff? 2. Does MiM Kids fit with your current work tasks and job description? **If no**: Why not? Can you please explain that a little bit? 3. How much of your time or effort is required for MiM Kids activities (e.g., per day or week)?    1. Has the amount of time or effort changed over time?   **If yes**: In what ways?   - 1. Have your responsibilities for MiM Kids changed over time?   **If yes**: How?   1. Do you feel that your role and work in MiM Kids is valued and recognized?    1. **If yes OR no**: Can you tell me more about why you feel that way? **Probe for:** *How* their job is recognized/valued; specific people that they feel do/don’t value their work; other examples |
| **Construct #4: Training, resources, and implemented activities** | 1. In your opinion, how successful were MiM Kids training sessions (School: EWKM/PH, After school: OSNAP, Child Care: IMIL)? 2. Are there any changes we need to consider as we train new staff ? 3. Did your staff have sufficient resources to implement the curriculum as outlined?    1. What changes (if any) would you make to the training or materials provided?   ***** SCHOOL SECTOR ONLY*****   1. Thinking about your school wellness committee, what obesity-related activities did the committee implement last year?   ***** ALL LEADERS*****   1. Did any activities in the past year focus on changes in policies or practices to promote physical activity, healthy eating, and optimal sleep in children? |
| **Construct 5: Successes and Barriers; time commitment** | 16. Thinking back over your experiences with MiM Kids over the past year, what do you think has been working well?   - 1. Do you have any successes to share?  1. What problems or challenges (if any) have you or the staff implementing MiM Kids experienced? 2. Are there any ways you feel that MiM Kids components could be redesigned to improve them and/or make them more feasible for you or your colleagues to successfully implement? |
| **Construct #6: Parent Engagement** | 1. Are families concerned and aware about childhood obesity? 2. To what extent are parents engaged in combating or preventing childhood obesity? 3. How, if at all, has parent’s awareness of and/or engagement in this issue changed since MiM Kids was launched? 4. What do you think is necessary to increase parent engagement and awareness of childhood obesity prevention? |
| **Construct #7: Changes over time** | 1. Have there been any major changes in your organization since MiM Kids started?   **Probe for**: changes in staffing, and positive and negative changes.   - 1. **If yes**: What changes have been made?   2. What implications (if any) have these changes had for the implementation of MiM Kids?  1. Tell me about other initiatives your organization has implemented in the past year to prevent and control childhood obesity?    1. Were these initiatives launched before or after MiM Kids was implemented?    2. At your organization: Do your initiatives dealing with childhood obesity complement the MiM Kids program or is there overlap? |
| **Construct #8: Linkage** | 1. Have you noticed any connection between MiM Kids activities within your organization and obesity prevention efforts within the broader community? 2. To your knowledge, have children who are overweight or obese been referred to other obesity prevention programs in your community (e.g. Healthy Weight Clinic, after school programs)? 3. As part of MiM Kids, do you interact with other sectors (e.g. school system, health clinics, after school, child care, parks and recreation) in the community?   **If yes:**   - 1. Which sectors do you interact with?   2. What challenges do you encounter when communicating with the other sectors? **Probe for:** What works well, what doesn’t? Why or why not? |
| **Construct #9: Media Competition**  **(School Personnel Only)** | 1. Have you heard of the MiM Kids School Media Competition where kids in schools and after-school programs created video, song, or rap or artwork (like a poster)?   **If yes:**   - 1. Was this competition implemented at your school?   **If yes:**   - 1. What are your thoughts about how it was implemented in your school?   2. What was the general impression of the media competition in your school? How about the broader community?   3. What benefits do you think the media competition had for schools and after-school programs in general? What about benefits for the students?   4. How well equipped did you feel to help students and schools participate in the competition? Please Explain.   5. What changes, if any, would you recommend in the process?   **If no:**   - 1. What were some reasons why it was not implemented at your school?   2. What was the general impression of the media in the broader community?   3. What benefits do you think the media competition had for schools and after-school programs in general? What about benefits for the students?   4. What changes, if any, would you recommend in the process? |
| **Construct #10: Closing** | 1. If you were giving the choice to be a part of MiM Kids again, would you choose to?   **If yes:** Why?  **If no:** Why not?   1. Is there anything I haven’t asked about MiM Kids that you think is important for me to know? |
