# Supplementary material for: Using School Staff Members to Implement a Childhood Obesity Prevention Intervention in Low-Income School Districts: the Massachusetts Childhood Obesity Research Demonstration (MA-CORD Project), 2012–2014
Source: Prev Chronic Dis. 2017 Jan 12;14:E03. doi: 10.5888/pcd14.160381 (PMC5234440; doi:10.5888/pcd14.160381)
Supplement: Supplementary file 3 [file 16_0381AppendixC.docx]

**Appendix C – Interview Coding Scheme**

| **Key Implementation Outcome** | **Definition** | **Questions to Answer w/**  **Qualitative Work**  **(n=23 interviews of teachers, nurses, administrators)** |
| --- | --- | --- |
| **Acceptability** | Perception of fit of MiM kids overall (focus on leadership/administration)  Was this considered a good idea? | Does MiM Kids fit with values/goal of school?  Did teachers feel this was their role?  Did leadership support this? |
|  |  |  |
| **Adoption** | Intention to try, initial decision  Who actually participated? | Barriers or promoters to getting MiM Kids started:   - Teacher trainings - Wellness champion hiring/training - School activities - Participation in the media competition |
|  |  |  |
|  |  |  |
|  |  |  |
|  |  |  |
| **Appropriateness** | Was MiM Kids good for the target audience? (teachers/kids)  Was this ideal to implement? | Were the MiM Kids materials appropriate?   - Teacher training - Curriculum - Materials, messages of MiM Kids - Other activities |
|  |  |  |
| **Feasibility** | Actual fit; relevance;  compatibility; suitability  Was this realistic to implement? | Barriers or promoters to doing MiM Kids:   - Teaching the lessons - Completing all the lessons - Encouraging other activities in the schools |
|  |  |  |
| **Fidelity** | How well was it implemented? | What adaptations/changes were made to:   - Trainings - Curriculum - Planned activities   Code anything related to:   - Quality of intervention - Quantity of intervention |
|  |  |  |
| **Implementation Cost** | Resources required to complete intervention (time, materials)  Did schools have what they needed to carry out intervention? | Code any mention of:   - Time - Materials - Money (for activities) |
|  |  |  |
| **Reach** | Reach of intervention  Any linkages in community?  More use of materials elsewhere in other classes or ages? | Mention of impact of MiM Kids on:   - Students - Parents - Other teachers/staff - Community |
| **Sustainability** | Maintenance; continuation;  durability; incorporation  integration; institutionalization;  sustained use  Will this intervention continue? | Mention MiM Kids:   - In the future (“next year”) - In policy changes (which will continue on) - Continuation of activities past study - Incorporation of activities into regular life |
