# Supplementary material for: Using School Staff Members to Implement a Childhood Obesity Prevention Intervention in Low-Income School Districts: the Massachusetts Childhood Obesity Research Demonstration (MA-CORD Project), 2012–2014
Source: Prev Chronic Dis. 2017 Jan 12;14:E03. doi: 10.5888/pcd14.160381 (PMC5234440; doi:10.5888/pcd14.160381)
Supplement: Supplementary file 4 [file 16_0381AppendixD.docx]

**Appendix D - Key Illustrative Quotes Obtained From Qualitative Interviews of School Staff Members Participating in MA-CORD in Massachusetts, 2012–2013 (n=23)**

| **Acceptability** | **Facilitators** | |
| --- | --- | --- |
|  | **Key constructs ^a^** | **Illustrative quotes** |
|  | Principal is a champion for healthy living  Existing wellness initiatives and policies (C1)  School nurses found the project fit well in their work tasks | “I think we have a principal that’s very proactive, all kinds of health and wellness initiatives.” (#39, C1)  “The school committee here has a major focus on health and nutrition. It’s one of our major goals for the school committee.” (#33, C1)  “MIM Kids fit does fit our department’s priorities. The messages that they want us to promote are very important to us.” (#26, C2)  “I’m pretty flexible, but I think a lot of what it deal with I have to deal with anyway in my role. It’s different from a math teacher who’s doing the role as a champion versus a school nurse doing the role. You know what I mean? Really for me, it’s not become overburdensome to me.” (#38, C2, school nurse) |
|  | **Barriers** | |
|  | **Key constructs ^a^** | **Illustrative quotes** |
|  | Pressure due to standardized testing or academic demands in district  New superintendent and administrative turnover (C2) | “Their priority on improving the staff professional development so we can improve the kids’ standardized test scores.” (#7, C1)  “We just got a new superintendent, and it’s all about the testing that we need to do periodically throughout the year. Those test scores have to show an increase, so, unfortunately, a lot of the programs that we were implementing before have kinda gone by the wayside, because we have to stick with the academics.” (#20, C2) |
| **Adoption** | **Facilitators** | |
|  | **Key constructs ^a^** | **Illustrative quotes** |
|  | Rapport between wellness champions and the staff | “The ones [wellness champions] that are willing to make the ask and have a good rapport with the teachers, and the teachers understand that this is very important for the kids . . . those are the schools that are having the most success implementing the project.” (#10, C2). |
|  | **Barriers** | |
|  | **Key constructs ^a^** | **Illustrative quotes** |
|  | Weather interrupted trainings (C2)  Lack of time for teachers to attend trainings  Teachers not informed about intervention (C2) | “We had a couple, I believe; a couple of [teacher training] sessions that had to be cancelled because of snow.” (#10, C2)  “The problem with the teacher training was just getting them there, cause there’s so much else on their plates.” (#10, C2)  “We [teachers] were unaware that the program had existed. It wasn’t really communicated to us until we went to the training . . . You were kind of on your own. That's our district as well. Whatever training we get, it's really not training. A lecture, here you go. Do it.” (#28, C2)  “I’ve always been confused. Just because there was so many things happening at the same time. . . I always was a little confused; who’s doing what?” (#36, C2) |
| **Appropriateness** | **Facilitators** | |
|  | **Key constructs ^a^** | **Illustrative quotes** |
|  | Training and curricula were well-received  Message appropriate for students  Teachers liked being part of a larger movement across schools | “It was really good training. . . I really left with the feeling like this was easy to implement and it would make an impact on the children right away, and they would be interested right away.” (#17, C2)  “I like that the lessons also tie in other subject areas like math, and science and language arts, I think that’s important, too.” (#1, C1)  “I think the messages chosen are easy concepts for our students to understand and they clearly get it right away.” (#26, C2)  “I liked the community that we—‘cause I seldom get to see the other teachers, so to get the feedback from the different schools and to know that I was actually doing similar things. It was encouraging to continue to even do more.” (#7, C1) |
|  | **Barriers** | |
|  | **Key constructs ^a^** | **Illustrative quotes** |
|  | Concerns about messages that children do not have control over | “I think some of the goals are lofty. I mean, we can promote sleeping. I think it’s important, but aside from educating, what more can you do? A lot of our kids are doubled up. Some of ‘em don’t even have beds; they’re in the living room. Family’s watching TV in the living room, the reality of their situation. There’s a fair number that are like that. Sleep is something that is chronically interrupted, and it has more to do with housing than anything else.” (#17, C2) |
| **Feasibility and Fidelity** | **Facilitators** | |
|  | **Key constructs ^a^** | **Illustrative quotes** |
|  | A champion at the school who maintains enthusiasm  Using students to engage other students  Technical assistance to change policies in the school | I think that having someone sort of in charge of that has helped a lot keeping it together. When someone—if there isn’t a presence sort of coordinating it, then it sort of falls apart. (#36, C2)  I mean, like I said, right now we have a great support in our principal, and that’s everything. If you don’t have the support from the administration, you’re not gonna have a program. (#28, C2)  I think what has worked really well is involving the students in the presentation of any of the topics, because we’ve been talking, have really come to the conclusion that they learn best from their peers and reinforce those things in their own language. Really kind of makes it hit home. (#40, C1)  “They are getting some serious technical assistance from a national organization that is helping them to identify best practices and resources that they can use to realize the wellness policy to the greatest degree possible in their buildings. I think that’s one of the key strategies.” (#10, C12) |
|  | **Barriers** | |
|  | **Key constructs ^a^** | **Illustrative quotes** |
|  | Competing priorities with teaching and with other campaigns  Principal and teacher turnover (C2)  Poor communication between staff | Right now, I know the teachers in my building are working from 7:00 a.m. to 6:00 p.m. It's all on academic stuff. (#28, C2)  We had to teach more than just PE last year. We had bullying classes to teach and there was just not enough time (#26, C2)  It’s been a very difficult couple of years. Lot of stress and lot of teachers don’t really have a lot of time to do some of these things that I think are very valuable. (#36, C2)  No, it's just that we have very busy days here. I, like I said, I could—I'm lucky that I can do 45 minutes to an hour per week out of really an 80 hour week. (#18, C1)  Six elementary and one middle school had a new principal this year. With the performance variance, we have a complete change in leadership, which really, I think, changes the focus because staff are looking to see, “Okay, what does this person need from me? What are the expectations? (#10, C2)  “We even waited months. I don't think we got any of the equipment—well, I think we got it, but I think it kinda sat with the principal or in the basement. For months we weren't aware of it.” (#28, C2) |
| **Implementation Cost** | **Facilitators** | |
|  | **Key constructs ^a^** | **Illustrative quotes** |
|  | Providing physical activity equipment to schools (C2) | Actually, I believe we actually got a grant from you last year that we utilized to provide more recess equipment that the children utilize so that they have more activities that they are involved in. We also use—we use some of the promotion materials that you’ve given us to try and keep their interest piqued for the physical activity, so to me, you’re a resource. (#38, C2)  We did get lots of playground equipment, like balls, hula hoops, jump ropes, different things like that for the kids. We put it by—the school was in suites, and most of the grades each have a suite. We dedicated certain supplies—age appropriate for those kids to use and bring out. We also have one large parachute that the kids—the teachers can request to bring out for their kids. (#25, C2) |
|  | **Barriers** | |
|  | **Key constructs ^a^** | **Illustrative quotes** |
|  | Inadequate printing resources to conduct lessons  Lack of media equipment for student activities related to intervention | That's the only thing, like I said, is sometimes our printers don't work at school. Sometimes there's no paper. (#24, C2)  “It would be nice to provide some video assistance and technology to families that don't have that, because those kids have wonderful ideas too but they don't have access to the technology at their house to create an infomercial. It would be nice if we could do that in school. That's something that we have limited equipment for as well.” (#18, C1) |
| **Reach** | **Facilitators** | |
|  | **Key constructs ^a^** | **Illustrative quotes** |
|  | School-wide integration of messaging  Linkages with other school health priorities  Media coverage  Children bringing messages home from school | *“*I think again I said that the five behaviors maybe easier for kids to understand it. I think that what’s working best is that it’s now being reinforced everywhere, within the health classrooms, within the cafeteria. It’s in the P.E. classes, so it wasn’t hard to get the messages out there. I think that’s what’s working best is that because it was simplified, now the kids see it everywhere, and it makes them more aware.” *(#1, C1)*  *“*That’s an advantage for the project because, for instance, the Oral Health Program, what presented an opportunity to disseminate the materials that were produced for parents for the Mass in Motion project, to convey the goals of the project and give parents some ideas of ways that they can meet those goals with their kids. . . We’re trying to reduce sugar-sweetened beverages, and the outcome for that is gonna be, not only reduction of obesity, but it’s also gonna reduce tooth decay. (#10, C2)  I do live right in the city, so I can see local cable. I’m pretty sure I saw a show that was talking about Mass in Motion. If I saw it, I’m sure other people in my community have seen it. That’s a good connection between what’s going on at school and what’s going on in the community. (#17, C2)  The feedback that we're getting from the parents is that by doing this in health class, the kids come home and they talk about it, and then they see it in the grocery store. Parents are now buying things that they themselves have never eaten because children are requesting it. (#18, C1) |
|  | **Barriers** | |
|  | **Key constructs ^a^** | **Illustrative quotes** |
|  | Limited collaboration between other sectors | *“*I think everybody’s just doing their own thing. Some of the kids at school will go to the YMCA for daycare, and then they do their own programs.” (#34, C2) |
| **Sustainability** | **Facilitators** | |
|  | **Key constructs ^a^** | **Illustrative quotes** |
|  | Health teachers or school nurses implementing curriculum  Enjoyable activities that are adopted long-term  Intervention involvement acknowledged in teacher evaluations | “I think that the people who are invested in health are involved. The health person that’s covering when they’re here—the most consistent one, and when they’re here they’re excited about it, and they wanna be a part of it. The teachers that are focused on the classes that are related to health and wellness, they’re excited to be a part of it. . .” (#42, C2)  We have a walking club. I do it every morning early on. A lot of these things have started or have continued because of the program. (#36, C2)  “I would be recognized by my principal, not so much my peers but my principal, for doing the work on the Mass in Motion that would count as part of the teachers' new evaluation system.” (#24, C1)  “This year, the expectations are higher. You have to have three goals, and you have to have activities surrounding that. You have to try and make it more sustainable, which is good. The expectations are higher.” (#42, C2) |
|  | **Barriers** | |
|  | **Key constructs ^a^** | **Illustrative quotes** |
|  | Staff turnover  Lack of ongoing leadership | “Some of the teachers have changed. We’ve had retirements, or people who have been let go, since Mass Kids in Motion. Some of them are brand-new. They don’t know anything about the program.” (#20, C2)  “I didn't even realize that it was an ongoing thing, so I think more advertising of it within our district would be helpful. I don't think there's enough awareness.” (#28, C2) |
| **^a^ Refers to both communities unless other specified**  **C1: Community 1, C2: Community 2** | | |
